# Graphene Nanoplatelet (GNPs) Doped Carbon Nanofiber (CNF) System: Effect of GNPs on the Graphitic Structure of Creep Stress and Non-Creep Stress Stabilized Polyacrylonitrile (PAN)

**DOI:** 10.3390/nano10020351

**Published:** 2020-02-18

**Authors:** Annas Bin Ali, Franz Renz, Julian Koch, Christoph Tegenkamp, Ralf Sindelar

**Affiliations:** 1 Institut für Anorganische Chemie, Leibniz Universität Hannover, Callinstr. 7, 30167 Hannover, Germany; franz.renz@acd.uni-hannover.de; 2 Hannover School for Nanotechnology, Laboratorium für Nano und Quantenengineering (LNQE), Leibniz Universität Hannover, Schneiderberg 39, 30167 Hannover, Germany; christoph.tegenkamp@physik.tu-chemnitz.de (C.T.); ralf.sindelar@hs-hannover.de (R.S.); 3 Faculty II, Hochschule Hannover, University of Applied Sciences and Arts, Ricklinger Stadtweg 120, 30459 Hannover, Germany; 4 Institut für Festkörperphysik, Leibniz Universität Hannover, Appelstraße 2, 30167 Hannover, Germany; koch@fkp.uni-hannover.de; 5 Institut für Physik, Technische Universität Chemnitz, Reichenhainer Str. 70, 09107 Chemnitz, Germany

**Keywords:** polyacrylonitrile, graphene nanoplatelets, stabilization, carbonization, shrinkage stress, creep stress, graphitization, electrical anisotropy

## Abstract

Improving the graphitic structure in carbon nanofibers (CNFs) is important for exploiting their potential in mechanical, electrical and electrochemical applications. Typically, the synthesis of carbon fibers with a highly graphitized structure demands a high temperature of almost 2500 °C. Furthermore, to achieve an improved graphitic structure, the stabilization of a precursor fiber has to be assisted by the presence of tension in order to enhance the molecular orientation. Keeping this in view, herein we report on the fabrication of graphene nanoplatelets (GNPs) doped carbon nanofibers using electrospinning followed by oxidative stabilization and carbonization. The effect of doping GNPs on the graphitic structure was investigated by carbonizing them at various temperatures (1000 °C, 1200 °C, 1500 °C and 1700 °C). Additionally, a stabilization was achieved with and without constant creep stress (only shrinkage stress) for both pristine and doped precursor nanofibers, which were eventually carbonized at 1700 °C. Our findings reveal that the GNPs doping results in improving the graphitic structure of polyacrylonitrile (PAN). Further, in addition to the templating effect during the nucleation and growth of graphitic crystals, the GNPs encapsulated in the PAN nanofiber matrix act in-situ as micro clamp units performing the anchoring function by preventing the loss of molecular orientation during the stabilization stage, when no external tension is applied to nanofiber mats. The templating effect of the entire graphitization process is reflected by an increased electrical conductivity along the fibers. Simultaneously, the electrical anisotropy is reduced, i.e., the GNPs provide effective pathways with improved conductivity acting like bridges between the nanofibers resulting in an improved conductivity across the fiber direction compared to the pristine PAN system.

## 1. Introduction 

Carbon fibers are by far the most prominent commercialized product in carbon and carbon reinforced materials. After nearly four decades of development, their properties regarding mechanical strength and electrical conductivity appear to have reached the saturation stage [1,2,3]. However, since the last decade, efforts are being made to employ nano-carbons as a reinforcement or property agent for an improved structure that would replicate into better mechanical, electrical and electrochemical properties. Especially the use of carbon nanofibers with average diameters of 100–300 nm as compared to carbon fibers (~7 μm diameter) are finding their ways into a variety of nano technological applications due to their high aspect ratio, increase of the surface area, high strength to weight ratio, improved charge transfer and greater possibility to modify the carbon chemistry [4,5,6]. 

Carbon nanofibers are typically fabricated via a top down approach by means of electrospinning followed by further steps for stabilization and carbonization or by a bottom up approach, using vapor growth processes. Electrospinning of polymer followed by stabilization in air and subsequent carbonization in a reduced atmosphere is not only a comparatively inexpensive and easy fabrication process, but it also offers a wide range of doping and chemical modifications to the made to polymer precursor in order to realize different functional properties [1,7,8]. Among various precursors known, polyacrylonitrile (PAN) is the most employed one for the production of carbon fibers [2,9]. In this top down approach, the cyclization process in the carbon nanofiber production is of extreme importance as during this step the formation of graphene-like ribbon structures take place. Without this process, a so-called ladder structure is avoided and PAN cannot withstand the carbonization process. The formation of the ladder like structure during the cyclization process prevents the fusion and loss of material at high temperature [8,10]. In general, the microstructure after carbonization is not highly graphitic and in order to increase the size and alignment of the graphitic domains, the fibers need to be graphitized at higher temperatures (2500 °C or more), which in turn increases the costs and energy consumption. Hence forth, the ability to improve the graphitic structure at lower temperatures is highly desirable with regards to economic and application perspective.

One of the approaches to improve the graphitic structure is to enhance the chain orientation inside the polymer precursor fiber during the stabilization process. During this process, tension is applied to the nanofibers to encounter the shrinkage of fibers, which otherwise reduces the polymer chain alignment. Recent studies have shown that the preservation and improvement of chain alignment is instrumental in producing highly graphitic aligned domains during the carbonization process, which in turn results in improved mechanical and electrical properties [11,12,13]. It is known further that the addition of various carbon allotropes (nanotubes, nanoparticles and graphene sheets) helps in improving the graphitization of carbon based films and nanofibers [5,14]. For example, single wall carbon nanotubes (SWCNTs) and multi walled carbon nanotubes (MWCNTs) have been incorporated in electrospun carbon nanofibers that results in improving the graphitization and crystallinity of PAN. Similarly, graphene nanoplatelets and graphene nano ribbons affect the crystallinity and graphitization of the polymer precursor [15,16,17,18]. Graphene nano platelets (GNPs), typically consisting of single to few layer sheets of sp^2^-hybridized carbon atoms forming two-dimensional layers with thickness in the nanometer scale, possess attractive characteristics such as high electrical conductivity, high modulus, high charge transfer capacity and high specific surface area [19,20]. As compared to chemical vapor deposition (CVD) grown carbon nanotubes (CNTs), the graphene platelets are comparatively cheaper. Additionally, dispersion of CNTs is tricky in a sense that it often gets curved inside the polymer matrix causing the grown graphitic layers to follow the curvature which results in a reduced graphitization [3]. GNPs used as dopants in carbon nanofibers can not only help in achieving high graphitic content at low temperatures, but also serve as anchors for polymer chains, thereby possibly reliving the use of external tension applied to nanofibers during the stabilization process. Only a very limited number of studies are available where the effect of GNPs on the graphtic structure of PAN has been investigated. One of the recent study done using PAN nanofibers doped GNPs revealed an increased degree of graphitization and an improved electrical conductivity developed by pressurized gyration followed by spark plasma sintering [21]. However, no complete study is available for electrospun carbon nanofibers doped with graphene platelets, especially regarding the templating effect of GNPs on PAN graphitic structure in presence and absence of externally applied creeps stress during the stabilization. In order to explore this, we have investigated how GNPs impact the graphitization degree in the absence and presence of externally applied creep stress during the complete cyclization process and investigated if the absence of creep stress be mediate the by presence of GNPs only.

In principal, the following routes can be followed for stabilizing PAN nanofibers: (1) application of an externally applied creep load, (2) mechanical fixing of nanofiber mats, where only the inherent shrinkage stress due to entropic relaxation is present, and (3) neither externally applied stress nor any mechanical fixing. The first two cases, imposing dimensional constraints and stretching along the fiber axis, is known to avoid entropic relaxation or any physical shrinkage. For the third case, since the PAN fibers are not constrained, the process results in loss of material during the carbonization step. For the present study, the first two cases are considered. We report on the development of PAN and GNPs doped PAN nanofibers via electrospinning, followed by stabilization in air and carbonization in an inert atmosphere. The effect of adding GNPs to the stabilized and graphitized PAN structure was investigated by Infrared Spectroscopy (IR), Scanning Electron Microscopy (SEM), Raman, X-ray diffraction (XRD) and X-ray photoelectron spectroscopy (XPS). To realize the effect of applying creep stress during stabilization, pristine and doped nanofiber mats were also stabilized with creep stress and carbonized at 1700 °C. The conductivity of fiber mats was subsequently measured for carbonized samples for both cases (creep stress stabilized and non-creep stress stabilized system). The presence of GNPs acts as templates and nucleating agents for an improved graphitic structure. GNPs engraved in nanofiber matrix performs as micro-anchors, preserving the polymer chain alignment and reducing the effect of shrinkage in absence of external stress during the stabilization.

## 2. Material & Methods

### 2.1. Materials

Polyacrylonitrile (PAN: average molecular weight 150,000 M_w_, Sigma Aldrich, St. Louis, MO, USA), dissolved in dimethylformamide (DMF, Carl Roth, Karlsruhe, Germany), was used as a precursor for carbon nanofiber production. Graphene nano platelets (GNPs) 6–8 nm thick and nearly 25 μm in width (ABCR Gmbh, Karlsruhe, Germany) were added as nano dopant. A mixture of 2% H_2_/98% Ar (Westfalen AG, Hannover, Germany) was used as medium for high temperature carbonization process. 

### 2.2. Synthesis of Carbon Nanofibers and Graphene Nanoplatelets (GNPs) Doped Carbon Nanofiber System 

**Figure 1:**
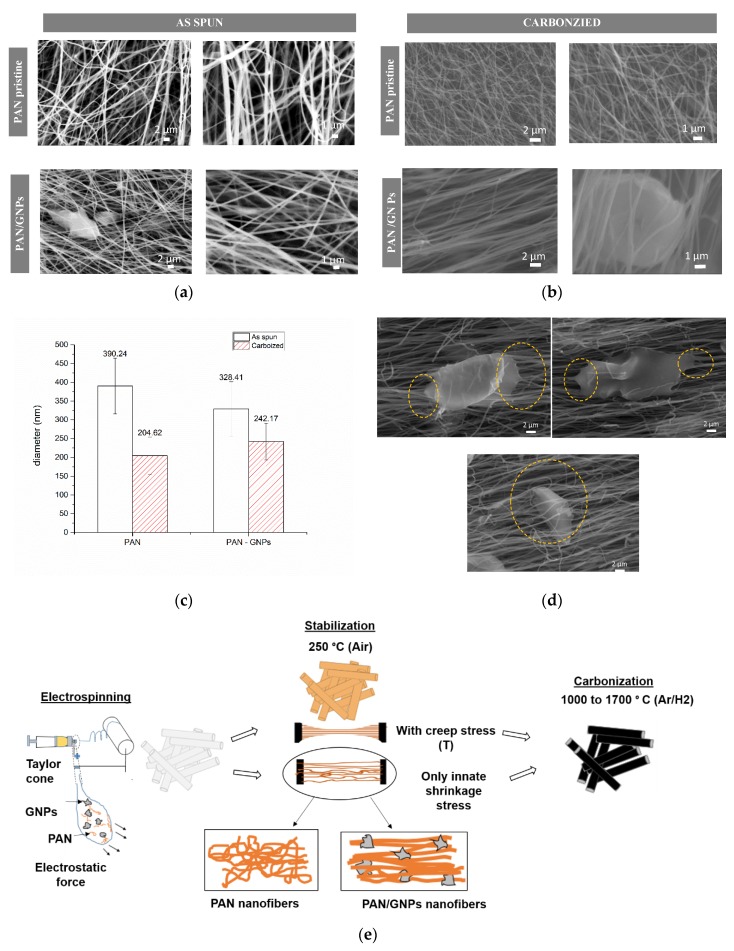
(**a**,**b**) Scanning Electron Microscopy (SEM) images for as-spun and carbonized pristine polyacrylonitrile (PAN) and PAN/graphene nanoplatelets (GNPs) doped nanofibers; (**c**) average diameter for as-spun and carbonized (1700 °C) PAN and PAN/GNPs nanofibers; (**d**) SEM images for carbonized PAN/GNPs nanofiber at 1700 °C showing graphene nanoplatelets (GNPs) engraved in nanofiber mats; (**e**) graphical abstract of polyacrylonitrile (PAN) and PAN/GNPs doped carbon nanofiber (CNF) showing fabrication process and two different ways in which stabilization is performed. GNPs helps in preserving the polymer chain alignment by acting as anchors and stretching the nanofibers.

Typically, the precursor solution for the spinning process was prepared by dissolving polyacrylonitrile (PAN) in DMF (13 wt%). The mixture was dissolved at room temperature overnight for duration of 12 h. For PAN doped GNPs system, GNPs (1 wt% by PAN) were repeatedly ultrasonicated for 1 h in DMF for 15 min, followed by a 15 min break to avoid agglomeration due to heating. Afterwards, PAN was added to it and dissolved similarly for 12 h. Both spinning solutions (pristine PAN and PAN/GNPs) were electrospun at 17 kV, 1 mL/h and 10 m/s collector speed, while the fibers were collected on an aluminum foil. Electrospinning was followed by stabilization and high temperature carbonization. Stabilization is performed at 250 °C for duration of 10 h in air for as spun PAN mats and PAN/GNPs in two different ways. For one set of the samples, the nanofiber mats were stabilized with gripping both ends of the whole mats to reduce the entropic shrinkage, while for the other set of samples, an additional creep stress of ~16 MPa was applied for the entire duration of the cyclization process. The latter samples are denoted with suffix ‘T’. PAN and PAN/GNPs were then carbonized under a mixture of 2% H_2_/98% Ar for duration of 1 h at four different temperatures (1000 °C, 1200 °C, 1500 °C and 1700 °C). Eventually, to observe the impact of creep stress, both PAN–T and PAN/GNPs–T were also carbonized at 1700 °C. The graphical abstract in Figure 1e sketches the complete fabrication process.

### 2.3. Characterizations

The morphology of as-spun and carbonized PAN and PAN/GNPs mats were analyzed using Scanning Electron Microscopy (SEM, Carl Zeiss LEO 1455VP, Oberkochen, Germany). For measuring the diameters of as-spun and carbonized PAN and PAN/GNPs, Image J was used. Fifty readings were taken from, in total, 10 different SEM images. Molecular structure of as-spun and stabilized mats was studied using Fourier Transform Infrared Spectroscopy (FTIR, Perkin Spectrum Two, Waltham, MA, USA). The ring cyclization index (RCI) was calculated in order to evaluate the amount of the cross-linked ladder polymer conversion by measuring the integral intensity of the C≡N (2240 cm^−1^) and C=N (1600 cm^−1^) peaks, ($RCI=\frac{I_{C=N}}{I_{C\equiv N}+I_{C=N}}$). Structural properties were analyzed using X-ray diffraction (XRD, Bruker D Phaser, Billerica, MA, USA). Diffraction patterns were recorded from 10° to 80° with a step size of 0.03° using Cu-K_α_ (*λ* = 1.5406). The interlayer spacing, *d* (002) was calculated using Bragg law, *d* = (*λ*/2·sin*θ*), where *λ* is the wavelength of the X-ray source and *θ* is the Bragg angle. The crystallite size was estimated using the Scherrer equation, *L_c_* = (*K*·*λ*/β·cos*θ*), where *K* is the shape factor (0.89 for *L_c_*). β is the full width at half maximum (FWHM) expressed in radians. The relative crystallinity for as spun PAN and PAN/GNPs nanofiber was finally calculated by the ratio of the area belonging to the crystalline PAN peak (~17°) and the total area, including the amorphous and crystalline portion. The graphitic structure and disorder within the nanofiber mats were characterized further using Raman spectroscopy (DXR2, Thermo Fisher, Waltham, MA, USA). Five spectra were recorded with a laser wavelength of 532 nm as an excitation source in the range of 1000–3000 cm^−1^ with a low incident laser power of 1 mW to avoid heating effects. The D- and G-bands in the spectra were deconvoluted using Lorentz fitting of the *I_D_* and *I_G_* integral ratios. Moreover, the crystal sizes *L_a_* (apparent crystallite size along the basal plane) of carbon nanostructures were estimated via *L_a_* (nm) = (2.4 × 10^−10^)·*λ^4^_laser_* (*I_D_*/*I_G_*)^−1^ [22]. The degree of hybridization was calculated from sp^2^ and sp^3^ fraction of the C1s photoemission spectrum taken with XPS using an Al-K_α_ emission. The kinetic energies were measured using a hemispherical analyzer with a pass energy of 20 eV. Including the Shirley background, the C1s spectra were fitted using symmetric Gaussian-Lorentzian sum functions under the constraint that the energy difference between the sp^2^- and the sp^3^-peak is 0.8 eV [23]. 

The electrical conductivity of the carbon nanofiber mats was measured by a 4-point probe method under ultra-high vacuum using a 4-tip STM/SEM system. The samples were cut into approximately 1.5 cm × 1.5 cm mats and were fixed to the sample holder using silver paste. Direction orthogonal and perpendicular to nanofiber axis was marked before for evaluating conductivity across and in fiber direction. The conductivity was calculated from the resistance R including the dimensions of the samples via σ·(S/cm) = *L*/*A_true_·R*, where L is the distance between the both ends. The effective area *A_true_* was calculated by dividing the liner density of fiber mats by the density of CNF. Thereby the CNF density is assumed to be ~1.7 g/cm^3^, similar to density of commercial PAN based carbon fiber. The average values were calculated from five different samples for each type of sample.

## 3. Results and Discussions

### 3.1. Morphology and Molecular Structure of As-Spun and Stabilized Polyacrylonitrile (PAN) and PAN/GNPs

**Figure 2:**
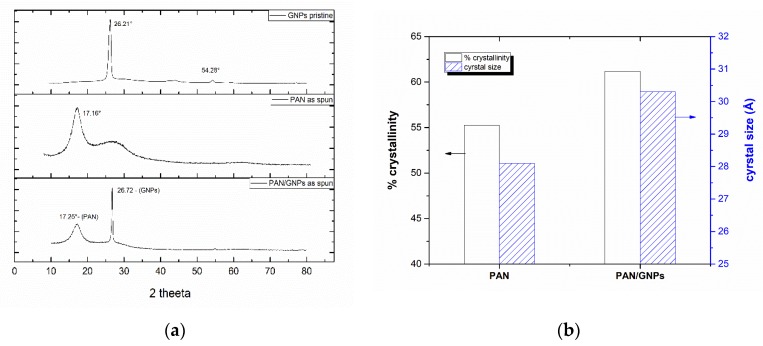
(**a**) X-ray diffractograms (XRD) for pristine GNPs, PAN and PAN/GNPs as-spun nanofibers; (**b**) crystallinity and crystal size for as spun PAN and PAN/GNPs nanofibers computed from XRD diffractograms.

Figure 1a,b shows SEM images of as-spun and carbonized nanofiber mats (non-creep stress stabilization). Both pristine PAN and PAN, doped with GNPs, showed a uniform and fibrous morphology. However, for PAN/GNPs, the formation of beads in some regions is observed due to changes of the viscosity and conductivity of the solution. Apparently, graphene sheets are beaded in both as-spun and carbonized PAN nanofiber mats. Furthermore, the average diameter for as spun PAN/GNPs has also decreased (Figure 1c), which can be attributed to the high conductivity of GNPs. This induces large charge accumulation in the solution jet which results in strong electrostatic repulsion among jet sprays. After carbonization at 1700 °C, the PAN and PAN/GNPs show a reduction in their diameters due to evolution of volatile species during the stabilization and carbonization reactions. Additionally, the entropic shrinkage during the cyclization also results in reduction of the diameter [8,9]. Interestingly, the average diameter reduction for PAN is 47% compared to 26% for PAN/GNPs nanofibers. This indicates that the addition of GNPs improves the thermal stability of PAN and reduces the shrinkage with comparatively less loss of material during the stabilization and carbonization processes. The effect of GNPs on PAN crystallinity was studied using XRD. The diffractograms for pristine PAN, GNPs and PAN/GNPs nanofiber mat are also shown in Figure 2a. PAN is a semi crystalline polymer showing a crystalline peak at ~17° corresponding to (100) crystallographic planes while a broad amorphous hallow at ~29° corresponds to (110), typical for semi-crystalline PAN. GNPs show a strong diffraction peak at ~26° corresponding to an interlayer spacing of 0.335 nm, which is close to that of graphite. The presence of a sharp peak indicates that not all GNPs are completely exfoliated. For the PAN/GNP composite both GNPs and PAN related peaks were found, i.e., the two phases coexist in as-spun PAN/GNPs nanofibers. Upon doping with GNPs, the amorphous portion is reduced and crystallinity of PAN increases. This high aspect ratio GNPs interact with polymer chains and organizes them in manner to improve the chain orientation which is reflected in an improved crystallinity and increased size of the PAN crystallites (Figure 2b).

**Figure 3:**
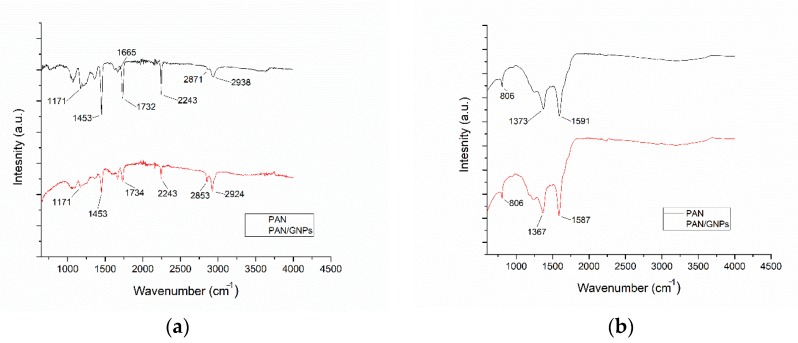
(**a**) Infrared (IR) spectra for as-spun PAN and PAN/GNPs nanofibers; (**b**) IR spectra for PAN and PAN/GNPs nanofibers stabilized at 250 °C for 10 h in air.

Characteristic IR spectra of as-spun and stabilized mats are shown in Figure 3a,b. The typical signature for PAN is the nitrile band (C≡N) at 2243 cm^−1^. The absorption observed at 2871 cm^−1^ and 2938 cm^−1^ corresponds to CH_2_ symmetric and asymmetric stretch modes, while other main intensity lines represent C=O stretch (1732 cm^−1^), C=C stretch (1665 cm^−1^) and C-O (1173 cm^−1^) vibrations. With the addition of GNPs, there is a small shift in C=O stretching and from 1732 cm^−1^ to 1734 cm^−1^ with a reduction in intensity, while the intensity line for 1663 cm^−1^ is increased. The changes in bands at higher wave numbers and especially at 1732 cm^−1^ hints towards an interaction of graphene platelets with the backbone C=O group. After stabilizing at 250 °C for 10 h, the evolution of the C=N band at ~1591 cm^−1^ takes place while the band at 2243 cm^−1^ is almost disappeared. This shows that the cyclization process has taken place and C≡N has been transformed to C=N, forming a ring like ladder structure. Ring cyclization index (RCI) showed the value of ~95% for both PAN and PAN/GNPs. The appearance of a peak at 806 cm^−1^ shows that the dehydrogenation has also taken place. For the cyclized mats, the shift in C=N can be observed which also points towards strong interaction of GNPs with PAN.

### 3.2. Carbonization and Graphitic Structure of PAN and GNPs Doped PAN

**Figure 4:**
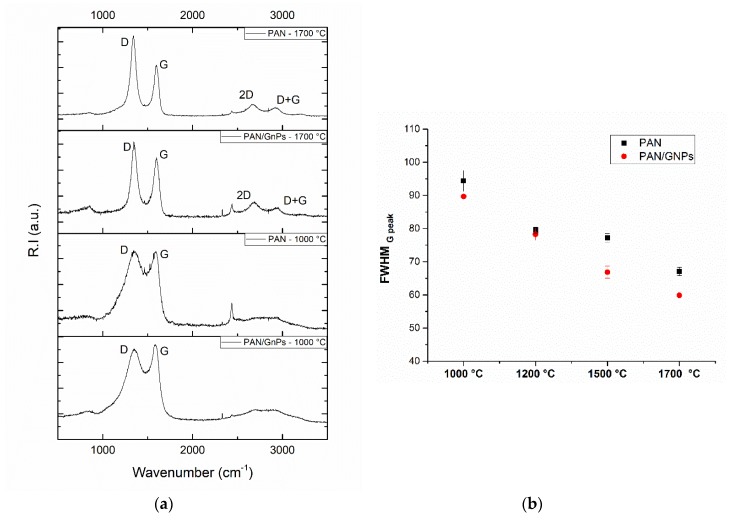

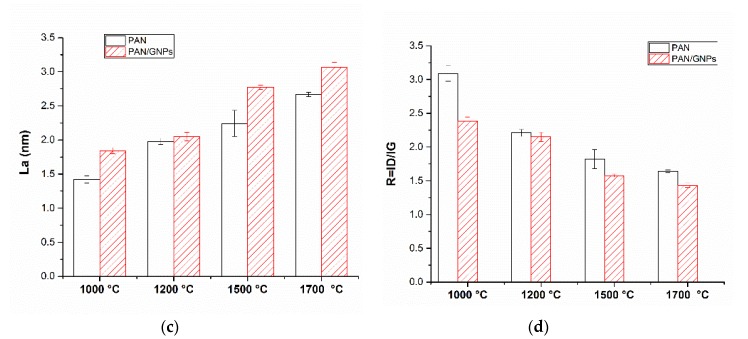
(**a**) Typical Raman spectra for PAN and PAN/GNPs carbonized at 1000 °C and 1700 °C; (**b**) FWHM_G-peak_; (**c**) *L_a_* and (**d**) *R* = (*I_D_*/*I_G_*) for PAN and PAN/GNPs doped nanofibers at different temperatures.

**Figure 5:**
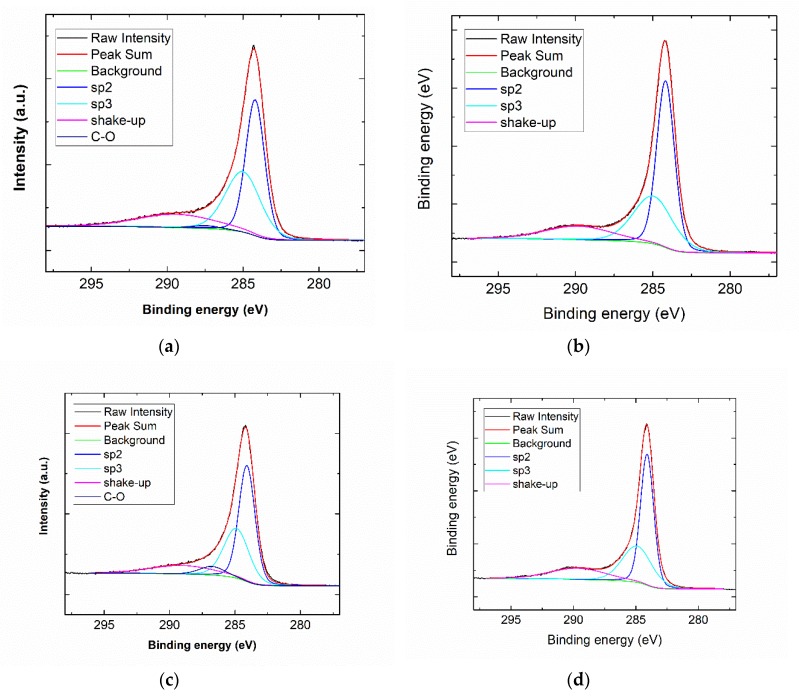
(**a**,**b**) Typical XPS spectra of de-convoluted C1s peak for pristine PAN and (**c**,**d**) PAN/GNPs at 1000 °C and 1700 °C.

The carbonization of non-creep stress stabilized PAN and PAN/GNPs were performed at four different temperatures (1000 °C, 1200 °C, 1500 °C and 1700 °C). To investigate the graphitic structure and crystallinity, Raman spectroscopy and XRD measurements were performed. Typical Raman spectra for the pristine GNPs, PAN and PAN/GNPs carbonized at 1000 °C and 1700 °C are shown in Figure 4a. The two major peaks centering nearly at 1360 cm^−1^ and 1585 cm^−1^ in the spectrum are referred to as D- and G-band. The G-band is attributed to an in-plane stretching mode of sp^2^ carbon bonds, while the D-band is due to hybridized vibrational mode related to the graphene layer edges, indicating the number of defects in the graphitic structure [22]. At lower temperatures of 1000 °C, the region in the range 2500–3000 cm^−1^ is majorly a hump with no sharp bands, however at higher temperature of 1700 °C the evolution of additional bands, 2D and D + D’ take place due to a two phonon process. The integral intensity ratio of D- and G-bands is indicative of the graphitic quality of samples and defects while the FWHM of G-band reflects the degree of graphitization [24]. As the temperature is increased, *I_D_*/*I_G_* ratio and FWHM of G peak decreases (Figure 4b,d). The FWHM of G peak decreases for PAN doped with GNPs (e.g., at 1700 °C; from 67.0 to 59.7 cm^−1^). The *L_a_* (domain crystal size along basal plane) shown in Figure 4c is also increased for GNPs doped PAN at all temperatures. All these changes are attributed to an enrichment of sp^2^ hybridized carbon and transformation of amorphous carbon into crystalline carbon. XRD results for PAN and PAN/GNPs are shown in Table 1. The FWHM of (002) peak decreases and the stacking size of crystal plane ‘*L_c_*’ increases (e.g., at 1700 °C, 2.03 nm for PAN and 2.47 nm for PAN/GNPs) which also suggests an increase of the carbon crystallinity. XPS was performed on pristine PAN and PAN/GNPs nanofibers at 1000 °C and 1700 °C. Typical XPS spectra for PAN and PAN/GNPs at 1000 °C and 1700 °C is shown in Figure 5. The sp^2^ content increases from nearly 55% to 59% upon doping with graphene platelets at 1000 °C and from 62% to 64% at 1700 °C (Table 2). The improved degree of graphitization supports the findings from Raman and XRD.

**Figure 6:**
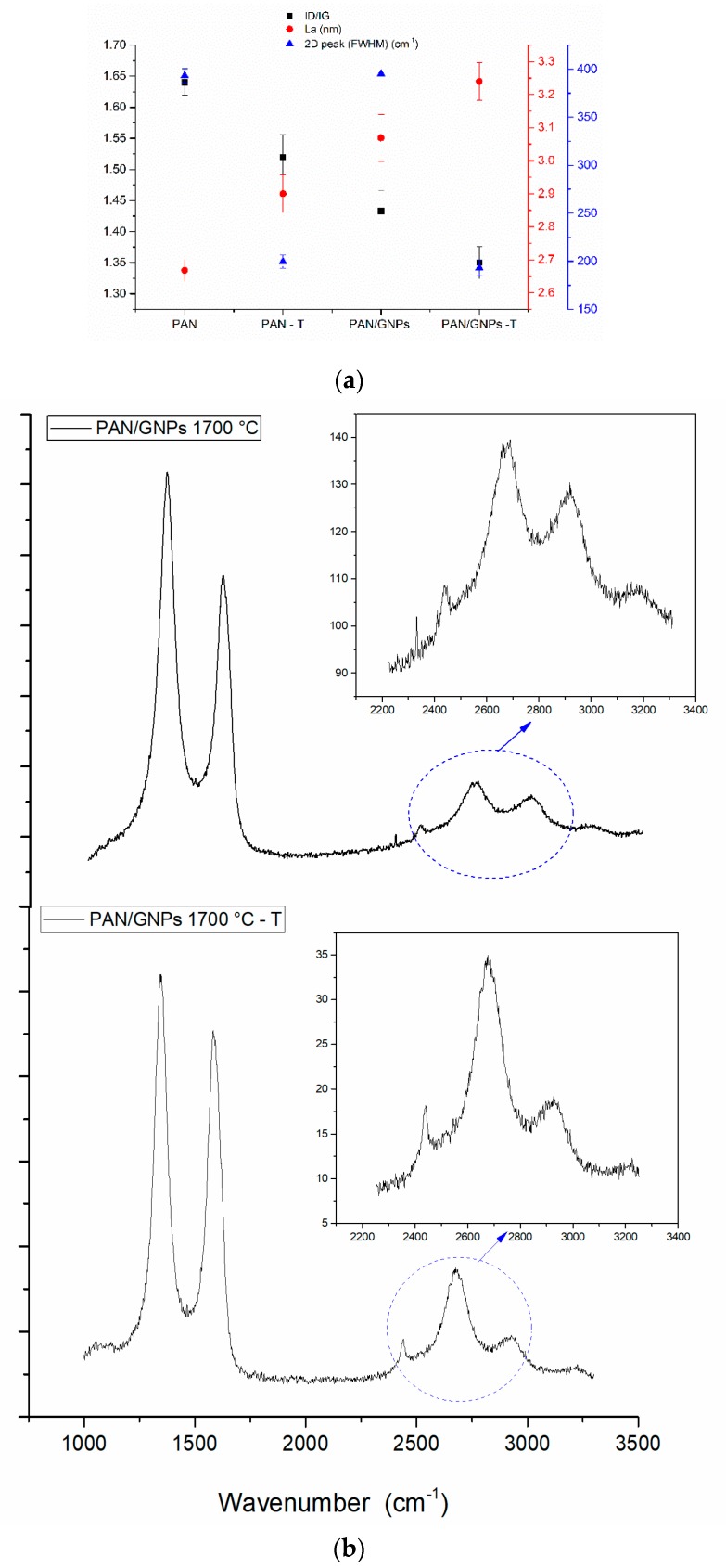
(**a**) *I_D_*/*I_G_*, *L_a_* and FWHM of 2D peak for spectra for PAN and PAN/GNPs stabilized with and without creep stress after carbonization at 1700 °C; (**b**) typical Raman spectra for PAN/GNPs stabilized with creep stress (PAN/GNPs 1700 °C–T) and without creep stress (PAN/GNPs 1700 °C) after carbonization at 1700 °C.

The above results are obtained for carbonized PAN and PAN/GNPs, which were cyclized by only fixing both ends of fiber mats (non-creep stress stabilized mats). To observe the impact of the creep stress, the carbonization was done at 1700 °C. The results of the Raman analysis are plotted in Figure 6a. It can be seen that the *I_D_*/*I_G_* ratio is further decreased and *L_a_* is increased for the samples stabilized with creep stress for both pristine PAN and PAN/GNPs. This shows that the graphitization is further enhanced and the crystallinity is increased. Moreover, it was found that the evolution of an intense and symmetric 2D peak takes place for ‘T’ samples (Figure 6b), while the FWHM of the 2D peak decreases (~195 cm) for both PAN–T and PAN/GNPs–T compared to (~370 cm) PAN and PAN/GNPs hinting towards ordering graphitic crystals. The 2D band is active due to a double resonance mechanism and is often used in general, for inferring the number of graphene layers for single, bilayer and multilayer graphene by calculation of *I_2D_*/*I_G_* ratio [24]. XRD results (Table 1) shows increased stacking size *L_c_* and decreased FWHM for the (002) peak.

The application of creep stress during the stabilization process not only helps to avoid the entropic shrinkage, but also ensures a high degree of chain alignment that prevents the curvature in carbon planes and eventually paves the way for an improved alignment of graphitic domains [11,25]. According to Haris et al. [26,27], the graphitizable carbons and non-graphitizable carbons differ in extent of fullerenic formation during the pyrolysis of polymer precursor. During the pyrolysis of PAN, the ribbon-like graphene ribbon is formed [28]. In these ribbons, along with the formation of six membered rings, the non-six membered heptane- and penta-rings fragments are also present. These majority of non-six membered rings fragments introduce a curvature in carbon plane which results in formation of a fullerenic structure. The introduction of curvature and curls in carbon planes prevents the long range stacking during the high temperature pyrolysis and is hindering the full graphitization process [29]. 

To summarize this part, both the diffraction and the Raman analysis showed that GNPs act as nucleation centers for the growth of graphitic carbon. The presence of GNPs promotes graphitization of PAN. Furthermore, even without the application of creep stress during the oxidative stabilization process, the PAN/GNPs doped fibers showed a greater extent of graphitization and crystallinity. In absence of external stress, the GNPs act as anchoring units for the alignment of PAN chains. From the SEM images (Figure 1d), it can be observed that these GNPs are embedded in PAN nanofiber mats and at discrete locations where they acts as possible fastening units that stretch PAN chains. These units of GNPs spun between the nanofibers prevent the loss of chain alignment during the stabilization process, which results in an improved degree of graphitization as compared to pristine PAN system. It is known, that during pyrolysis the polymer backbone serves as a nucleation point for growth of graphene ribbons [30]. The stronger interaction of GNPs with the PAN backbone supplemented by mechanical action of these GNPs in inducing stress in PAN chains is instrumental in preventing the curvature in carbon planes and maintaining long range order, which translates into improved graphitization of PAN. As evident, the sample cyclized under creep stress showed improved graphitization compared to non-creep stress cyclized samples, both for PAN and PAN/GNPs.

### 3.3. Electrical Conductivity of Pristine and GNPs Doped PAN Nanofiber Mats

**Figure 7:**
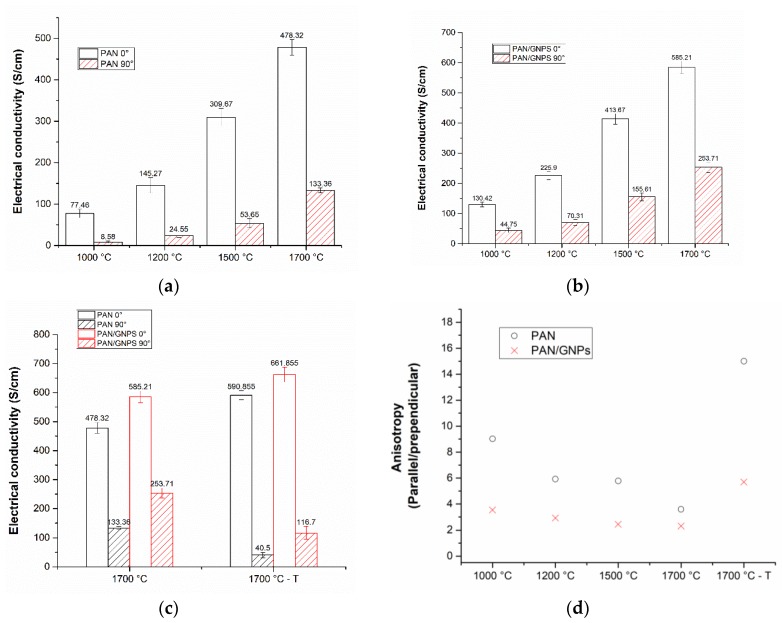
(**a**) Electrical conductivities of (**a**) PAN and (**b**) PAN/GNPs nanofiber mat after carbonization at different temperatures in parallel and perpendicular direction; (**c**) electrical conductivities of creep stress stabilized and non-creep stress stabilized PAN and PAN/GNPs nanofiber mats at 1700 °C in parallel and perpendicular directions; (**d**) anisotropy in electrical conductivity of carbonized PAN and PAN/GNPs.

**Figure 8:**
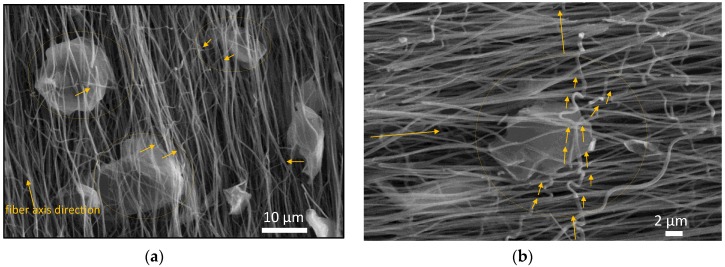
(**a**,**b**) SEM Images showing the bridging effect of GNPs between the individual nanofibers that mediates the presence of path ways for electrical conductivity in perpendicular.

The results of electrical conductivity measurements of PAN and PAN/GNPs are shown in Figure 7a,b. The transport was measured along and across the nanofiber axis direction for both types of samples. It can be seen that for all temperatures, doping with GNPs enhances the conductivity along the fiber direction. At 1000 °C, the electrical conductivity is increased by almost 60% along the fiber axis. Similarly, a 24% increase in electrical conductivity was observed at 1700 °C, suggesting that microstructure becomes more graphitic as supported by Raman and XRD results. A greater increment in conductivity at lower temperatures as compared to higher temperatures upon doping with GNPs can be attributed to the fact that the templating effect of nano-carbons is more pronounced at lower carbonization temperatures where a large portion of carbon is more amorphous [31]. Conductivity across the fiber directions is lower as compared to in fiber direction (e.g., at 1000 °C from average of 77 S/cm in fiber direction to 8 S/cm across the fiber direction). Such a large discrepancy in electrical conductivities across and along fiber direction is obvious as there are fewer interconnects available for electrons. Fibers make occasional contacts even when they are not stretched however they are aligned in one direction due to use of rotating collector after spinning. Interestingly for PAN/GNPs, the anisotropy in electrical conductivity is decreased as compared to pristine CNF. In the case of pristine CNFs, there are fewer paths for electron flowing in the perpendicular direction, however with incorporation of GNPs into CNFs, there are more conductive network like pathways across nanofibers. Uniformly distributed GNPs in fiber matrix at different locations provide a path due to which the anisotropy in conductivity is reduced as compared to pristine CNF (Figure 8). The anisotropy of the electrical conductivity is much higher for 1700 °C–T as compared to 1700 °C samples (Figure 7d). The average electrical conductivity parallel to fiber direction is 590.8 S/cm, however, across the fiber direction is 40.5 S/cm for PAN–1700 °C–T, i.e., 15 times lower. Similarly, a high anisotropy in electrical conductivity was reported earlier by us for creep stress cyclized PAN system [32]. For PAN/GNPs–1700 °C–T, the anisotropy in electrical conductivity is greatly reduced compared to PAN–1700 °C–T. The above finding suggest that the presence of GNPs triggers the formation of a 2D electrically conductive network by interfacial contacts between different fibers. GNPs acts as a bridge between the conductive fiber lines, not only by themselves, but also a network of nanofiber cross connections was observed where the GNPs are embedded in nanofibers (Figure 8a,b). Due to this, the conductivity is fairly enhanced across the fiber direction providing electrical paths. Carbon nanofibers are bridged across the nanofiber alignment direction where GNPs are present. It is important to mention here that the conductivity of carbon nanofiber mats or assembles cannot be directly interpreted as the electrical conductivity of single carbon nanofiber due to the fact that in addition to the conductivity of a single nanofiber, the entanglements and interconnects between the nanofibers also influence the electron flow. Our earlier studies have reported that the cross connections act as scattering centers for electron flow, details of which can be looked over [32].

In conventional carbon fiber fabrication process, the improved graphitization degree is closely associated with orientation of polymer chains in PAN precursor fiber. Carbon fibers are stabilized under constant tension in order to avoid the relaxation in amorphous region before carbonization. Stabilization under constant tension has proven to be an effective technique to reduce the entropic shrinkage and prevent the loss of polymer chain alignment especially in the amorphous region. It is interesting to observe that by adding GNPs, high graphitic content can be achieved even with absence of tension applied during the stabilization process. GNPs beaded carbon nanofiber can not only help to counter the absence of external tension as they anchors the polymer chain and prevent loss of chain orientation but also serve as templating agent, providing nucleation sites for growth of graphitic crystal. In other words, the GNPs embedded in nanofiber matrix can pre-organize the polymer chain into aligned structures which translates into highly orientated graphitic crystallite during carbonization.

## 4. Conclusions

In this study, pristine CNFs and GNPs doped CNFs were prepared via electrospinning followed by oxidative stabilization and carbonization. Stabilization at 250 °C for 10 h was performed without application of creep stress but with mechanical fixing of nanofiber mat ends for both pristine PAN and PAN doped with GNPs at four different carbonization temperatures. To observe and compare the effect of incorporated creep stress in the presence of nano-carbon doped PAN, the stabilization was also performed with the application of creep stress for PAN and PAN/GNPs at 250 °C for 10 h. These samples were carbonized at 1700 °C. The microstructure, graphitization and electrical conductivity was systematically investigated for these sets of samples. Our results revealed that the doping of CNFs with GNPs improves the crystalline structure and graphitization degree of polyacrylonitrile as confirmed by the decrease of the *I_D_*/*I_G_* ratio, FWHM for G peak and increased crystallite size *L_a_* and at all carbonization temperatures. GNPs are instrumental in providing bi-fold advantage, (1) doping GNPs enhances the graphitization of PAN by a templating effect of nano-carbon, (2) the presence of GNPs even during the absence of externally applied creep stress helps to maintain a certain degree of stress during the cyclization process which translates into improved alignment of graphitic domains resulting in higher degree of graphitization. GNPs in-situ act as stress units, engraved in nanofiber mats, which to reduce the shrinkage, in case no tension is applied. Furthermore, during the electrospinning process, additional electrostatic force acts on polymer solution jet due to GNPs which exerts shear to the polymer chains, resulting in prior alignment which translates into improved ordering during carbonization process. The application of creep stress in both PAN and PAN/GNPs result in an improved graphitization of the polymer. The transport experiments reveal highly anisotropic conductivities for PAN and PAN–T compared to PAN/GNPs and PAN/GNPs–T samples in perpendicular and parallel to fiber direction. GNPs, embedded in nanofibers, bridge the nanofibers across the nanofiber direction and forms a conductive network across the fiber alignment direction due to which the conductivity in perpendicular direction is increased. In absence of GNPs, there are fewer paths for electrical current flow across the fiber alignment direction and electron flow is impeded. The graphene nanoplatelets in continuous carbon nanofiber can provide a unique chemical structure with highly conductive network due to excellent 2D nanostructure and electrical conductivity of GNPs. Further doping schemes will be worked out in the future in order to produce porous carbon nanofibers beaded with GNPs that can be stabilized without a tension yet still able to provide with appreciable degree of graphitization at lower carbonization temperatures. This could lead to a production of highly conductive electrodes for energy storage and conversion still with porosity and defects which otherwise is a compromise, as defects and porosity undermines electrical transport.

## Figures and Tables

**Table 1:** The full width at half maximum (FWHM) _(002)_ and the crystallite size ‘*L_c_*’ for PAN and PAN/GNPs carbonized at different temperatures, as calculated from XRD results.

Sample	L_c_ (nm)	FWHM _(002)_
PAN–1000 °C	1.38	5.82
PAN–1200 °C	1.96	5.01
PAN–1500 °C	2.01	4.65
PAN–1700 °C	2.03	3.98
PAN/GNPs–1000 °C	1.68	5.14
PAN/GNPs–1200 °C	2.2	3.64
PAN/GNPs–1500 °C	2.33	3.45
PAN/GNPs–1700 °C	2.47	3.25
PAN–1700 °C–T	2.07	3.88
PAN/GNPs–1700 °C–T	2.58	3.12

**Table 2:** sp^2^ fraction and sp^2^/sp^3^ ratio for PAN and PAN/GNPs at 1000 °C and 1700 °C, as evaluated from de-convolution of C1s spectra for X-ray photoelectron spectroscopy (XPS) results.

Sample	sp^2^/sp^3^ Ratio	sp^2^ Fraction
PAN–1000 °C	1.22	54.8%
PAN/GNPs–1000 °C	1.50	59%
PAN–1700 °C	1.57	61.89%
PAN/GNPs–1700 °C	1.81	64.45%
